# Effect of Mesoporosity on Structural, Textural, and Optical Characteristics of Fe(III) Ion-Exchanged ZSM-5 Zeolites

**DOI:** 10.3390/molecules31010023

**Published:** 2025-12-22

**Authors:** Irina A. Zvereva, Azamat Samadov, Sergey A. Kurnosenko, Sergey O. Kirichenko, Marina G. Shelyapina, Vitalii Petranovskii

**Affiliations:** 1 Department of Chemical Thermodynamics and Kinetics, Institute of Chemistry, Saint Petersburg State University, 7/9 Universitetskaya Embankment, Saint Petersburg 199034, Russia; irina.zvereva@spbu.ru (I.A.Z.); azamatsamadov1245@gmail.com (A.S.); s.kurnosenko@spbu.ru (S.A.K.); 2 Centre for Innovative Technologies of Composite Nanomaterials, Saint Petersburg State University, 7/9 Universitetskaya Embankment, Saint Petersburg 199034, Russia; sergey.kirichenko@spbu.ru; 3 Department of Nuclear Physics Research Methods, Faculty of Physics, Saint Petersburg State University, 7/9 Universitetskaya Embankment, Saint Petersburg 199034, Russia; 4 Centro de Nanociencias y Nanotecnología, Universidad Nacional Autónoma de México, Ensenada 22800, BC, Mexico; vitalii@ens.cnyn.unam.mx

**Keywords:** zeolite, MFI, mesoporosity, Fe(III) ion-exchange, alkaline etching, iron nanospecies, bandgap energy

## Abstract

This study investigates the influence of mesoporosity, pre-created by alkali etching in ZSM-5 zeolite, on the characteristics of Fe^3+^ ion-exchange and subsequent changes in its textural and optical properties. It is shown that the formed hierarchical porosity facilitates the penetration of hydrated iron complexes into the internal channels. This not only increases the degree of exchange, but also leads to the formation of multinuclear Fe_x_O_y_ clusters and, possibly, to the partial isomorphic replacement of Al^3+^ with Fe^3+^ in the framework. Comprehensive characterization of mesoporous samples (XRD, SEM, N_2_ adsorption, UV-Vis) confirms the preservation of the microporous crystal structure of MFI on the one hand, and demonstrates a significant change in the distribution of iron-containing species in mesoporous matrices on the other. The introduction of Fe ions significantly reduces the bandgap energy, shifting the absorption edge into the visible range. The results obtained demonstrate that preliminary mesostructuring is an effective approach for creating hierarchically porous Fe zeolites with great potential for photocatalytic applications.

## 1. Introduction

Zeolites are industrial heterogeneous catalysts widely used in many reactions important for sustainable development [1,2,3]. Their catalytic activity can be tailored in various ways, including by adjusting Brønsted and Lewis acidity [4,5], by changing the Si/Al ratio [6], creating extra-framework Al during synthesis by selecting an appropriate organic structure-directing agent [7], passivating surface acidity in core–shell structures [8], introducing active sites through post-synthetic treatment [9,10], etc. Ion-exchange is a common and simple tool widely used to introduce metal species such as Cu, Ag, Fe, etc., into zeolite, which act as Lewis acid sites [11,12]. The target characteristics of the zeolite-based material, as a catalyst or a sorbent, are dependent of the ion-exchange conditions, including the solution pH, solution concentration, ion-exchange temperature and time, ion-exchange cycles, and so on [13]. Zeolites with ion-exchanged iron have attracted considerable attention due to their redox properties and wide operating temperature range in de-NOx reactions [14,15,16]. In addition, Fe-containing zeolites exhibit photocatalytic activity that is strongly associated with Fe-containing species [17]. The formation of dihydroxybenzenes during photocatalytic hydroxylation of phenol on an Fe-MFI catalyst is attributed by the authors in [18] to the active centers of isolated Fe^3+^ ions coordinated tetrahedrally. The authors in [19] relate the photocatalytic hydroxylation of phenol on Fe-MFI with tetrahedrally coordinated isolated Fe^3+^ ions. Recent studies have shown that the latter are also effective in Fenton-like degradation reactions [20].

Several studies reports that Fe^3+^ may substitute Al^3+^ in the frameworks of various zeolites, MOR, *BEA, FER, MEL, MFI, and FAU (here we use a three-letter coding system adopted by the International Zeolite Association [21]), creating new active catalytic sites [18,22,23,24,25,26,27,28,29,30,31]. Several publications confirm that Fe^3+^ can replace Al^3+^ in the MFI structure during hydrothermal synthesis [23,31]. The isomorphous substitution modifies the nature of acid sites and affects the neighboring atom distributions (both framework and extra-framework) [32]. For MEL zeolite, it was shown that the substitution of Al with Fe in the zeolite framework improves hydrophobicity and catalytic oxidation ability, making these materials potentially active in wet environments [28]. However, the degree and stability of such substitution depend on the synthesis conditions and material composition.

The state of iron species in zeolite, which significantly affects the properties of the host zeolite, is determined both by the method of introduction and processing conditions, as well as by the properties of the parent zeolite, such as acidity and porosity [33]. However, since the Fe^3+^ ion has a large hydration shell, it penetrates into zeolite pores with difficulty, that impedes rapid ion-exchange [22].

In recent years, there has been considerable interest in studying the effect of mesoporosity on the properties of zeolites [34,35,36,37,38,39]. In cases where bulky molecules act as reactants or reaction products, the reaction kinetics are very limited. The introduction of secondary porosity and its regulation can be viewed as a new parameter that opens up a huge field for the creation of new functional zeolite materials. The microporous zeolite framework is responsible for selectivity of the reaction, while mesopores enhance mass transfer and reaction kinetics [40,41,42].

To introduce secondary porosity into zeolites (mesopores (from 2 to 50 nm) or macropores (larger than 50 nm)), two main strategies have been developed: (i) modification of zeolite porosity by dealumination or desilication, in which zeolites are subjected to additional processing in an alkaline or acidic medium that leads to desilication [34,43,44] or dealumination [44,45,46] of the zeolite lattice (etching), and, as a result, the creation of disordered secondary porosity; (ii) synthesis of layered zeolites in the presence of organic structure-directing agent that can be followed by pillaring with different oxides, such as SiO_2_ [47,48], Nb_2_O_5_ [49], or TiO_2_ [50]. More information about various strategies to create zeolites with hierarchical porosity, and their advantages and drawbacks, can be found in several comprehensive reviews [1,41,51].

The main advantages of alkali etching are its simplicity, speed, and low cost [34]. The size of the created mesopores can be controlled by the processing time and/or temperature, the concentration of the solution, as well as the selection of the Si/Al ratio of the initial microporous zeolite [34,52]. Despite the simplicity and cheapness of creating zeolites with hierarchical porosity by leaching, this method leads to the formation of disordered secondary porosity, which may be a limiting factor for the reaction rate.

In this contribution, we report on the synthesis and comprehensive study of a series of new iron-containing MFI zeolites. They were obtained by treating commercially available microporous NH_4_-ZSM-5 zeolite (MFI framework) with an alkaline NaOH solution to create secondary porosity, followed by ion-exchange of these samples in a solution containing trivalent iron ions. The aim of this work is to shed light on the effect of mesoporosity on the accessibility of zeolite voids for hydrated Fe^3+^ complexes during the exchange process and, as a consequence, on the formation of various types of iron oxide clusters and their optical properties that are important for photocatalytic applications.

## 2. Results and Discussion

Three zeolite matrices were used for iron support: the commercial microporous NH_4_-ZSM-5 zeolite, labeled as Z0, and two mesoporous ZSM-5 matrices obtained by treatment of Z0 in 0.2 M or 0.4 M of NaOH aqueous solution, labeled as Z0.2 and Z0.4, respectively. The iron-containing samples were prepared by ion-exchange treatment of the zeolite matrices in 0.1, 0.5, or 1.0 N of FeCl_3_ aqueous solution. Hereafter in this paper, samples will be referred as Z*X*-Fe*Y*, where X indicates the concentration of the NaOH solution in the pre-treatment procedure and *Y* denotes the concentration of FeCl_3_ solution (example: Z0.2-Fe0.5). More details on the synthesis procedure can be found in Section 3.

### 2.1. Elemental Analysis

The overall elemental composition was determined by energy-dispersive X-ray fluorescence spectroscopy (EDXRF). EDXRF, due to its accuracy, rapidity, and multielement capacity, is becoming widely used in elemental analysis of zeolites [53,54,55]. Its penetration depth is between a few and hundreds of μm and for submicron particles can be used for probing bulk composition. The surface composition was probed by X-ray photoelectron spectroscopy (XPS). The results of the study of micro- and micro/mesoporous samples before and after the ion-exchange procedure are listed in Table 1.

In the parent material, Z0, the Si/Al ratio is essentially lower than the manufacturer’s nominal value, and the distribution of aluminum throughout the sample depth is uneven. The treatment in NaOH leads to Si leaching out (which creates mesoporosity as will be discussed later) and a more uniform distribution of Al. These effects are more pronounced for sample Z0.4, which was processed in a more concentrated solution. It should be noted that alkaline treatment of the parent ammonium form of ZSM-5 zeolite, along with the removal of Si, also results in the replacement of [NH_4_]^+^ with Na^+^. The excess of Na^+^ compared to the ion-exchange capacity may be associated with the formation and incomplete washing of additional sodium containing species, mainly in the formed mesopores and on the surface.

During ion-exchange of cations (residual ammonium and sodium introduced during alkaline etching) for iron ions (samples Z0-Fe0.1, Z0-Fe0.5, Z0-Fe1.0), there is a slight decrease in the Si/Al ratio (in volume, see Table 1, EDXRF data). However, the iron content depends on the sample selected as the precursor and increases in line with the intensity of the sample’s preliminary processing in NaOH. Assuming that one Fe^3+^ cation substitutes three [NH_4_]^+^ (and/or Na^+^) cations, the degree of ion-exchange for the minimally etched sample Z0-Fe0.1 can be estimated as 63%. However, for samples Z0.2-Fe0.1 and Z0.4-Fe0.1, which underwent more intensive alkaline etching, the situation changes dramatically during ion-exchange with iron: the Si/Al ratio, both on the surface and in the volume, increases compared to the parent Z0.2 and Z0.4 samples, which indicates a decrease in Al content. If we assume that the negative charge of the zeolite framework is compensated by all ions (Na^+^ and Fe^3+^), then there is a significant excess of positive charge. This suggests that either a part of Fe^3+^ substitutes framework Al^3+^, or some charged Fe_x_O_y_ species are formed. Ion-exchange in a more concentrated FeCl_3_ solution results in a subsequent increase in both the Si/Al and Fe/Al ratios, with the latter increasing sharply. Overall, this suggests further substitution of framework Al by Fe in the structure.

### 2.2. Structure and Morphology Studied by XRD and SEM

**Figure 1:**
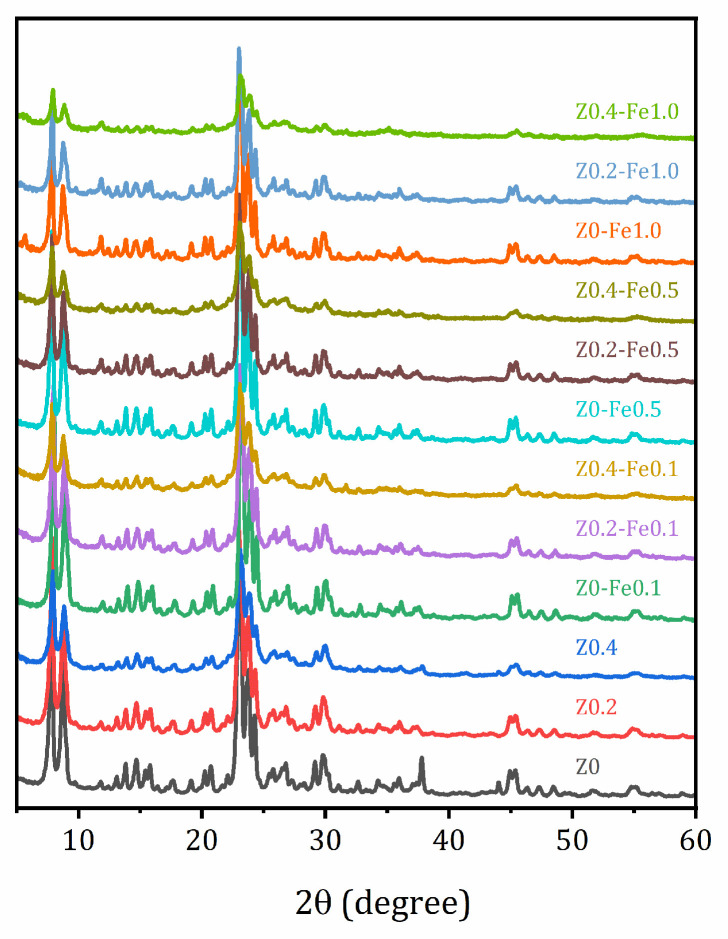
Powder XRD patterns of the studied samples.

The XRD patterns of the parent sample and the samples after alkaline treatment and ion-exchange are shown in Figure 1. They confirm that all the samples studied retain the MFI crystalline structure. However, alkaline treatment leads to the elimination of impurity peaks not associated with the MFI structure (at 37.8 and 44.0 degrees 2θ) and to the smoothing of the reflection peaks due to appearance defects caused by desilication. Ion-exchange with iron does not have a significant effect on the XRD patterns, and no new peaks appear.

**Figure 2:**
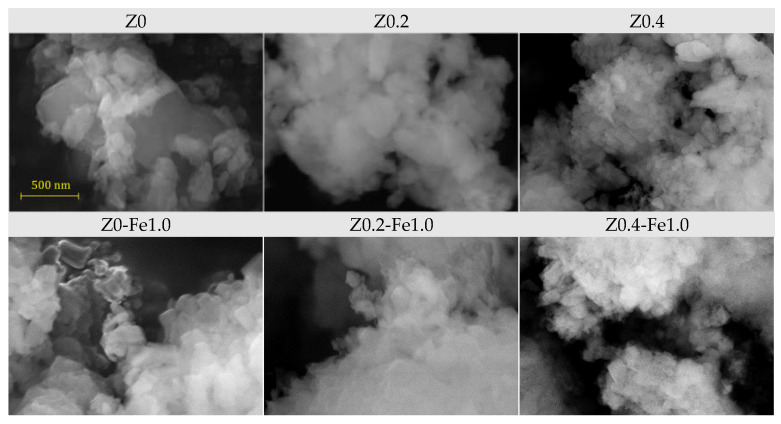
SEM images for the parent samples (**top-row**) and after ion-exchange in 1.0 N FeCl_3_ (**bottom-row**).

The morphology of the samples was studied by scanning electron microscopy (SEM). SEM images of the parent zeolite and samples after the treatment in NaOH, both before and after ion-exchange in the most concentrated FeCl_3_ solution (1.0 N), are shown in Figure 2. The morphology of the parent Z0 material can be described as individual crystals in the form of plates measuring approximately 100–500 nm in size, combined into agglomerates of several microns.

Alkaline treatment leads to a decrease in both the size of individual crystallites and the refinement of agglomerates; this effect becomes more pronounced with increasing concentration of the alkaline solution. Moreover, the shape of the crystallites becomes smoother. It is possible that a decrease in both the size of the crystallites and the size of the agglomerates, as well as a large number of surface defects due to the partial removal of silicon from their crystallographic positions, facilitates the replacement of aluminum with iron in the zeolite lattice. Ion-exchange with Fe^3+^ does not have a significant effect on the morphology of the samples, even at high concentration of the FeCl_3_ solution.

### 2.3. N_2_ Adsorption/Desorption

**Figure 3:**
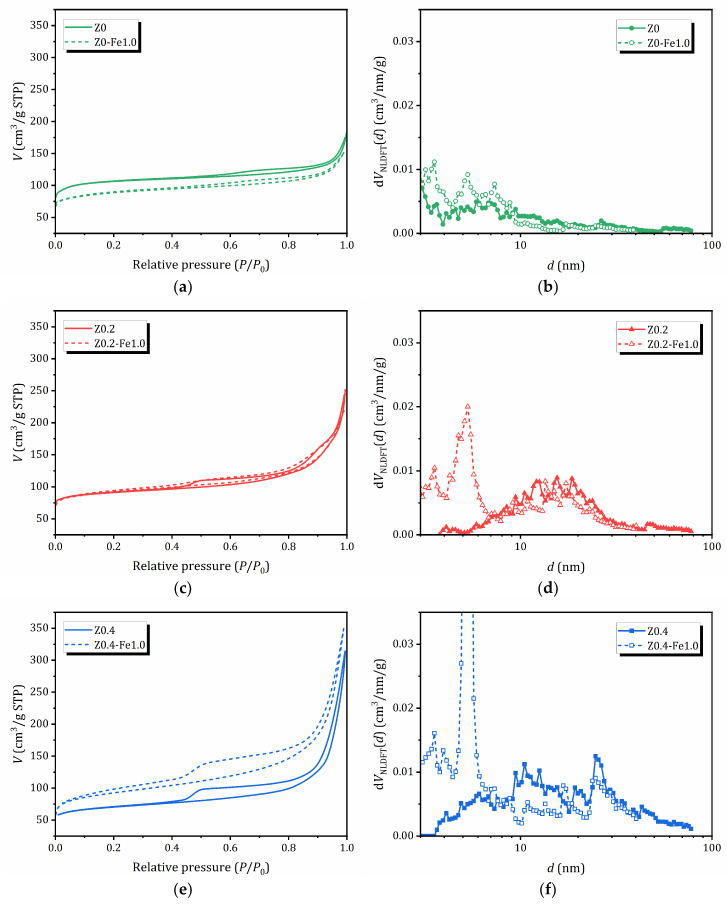
N_2_ adsorption/desorption isotherms (**a**,**c**,**e**) and NLDFT pore size distribution (**b**,**d**,**f**): a comparison between samples before and after treatment in 1.0 N FeCl_3_ solution.

The adsorption/desorption isotherms of N_2_ (77 K) for samples before and after treatment in a 1.0 N FeCl_3_ solution, as well as the pore size distribution evaluated by the nonlocal density functional theory (NLDFT) method using the QuadraWin program (Version 5.11) in a cylindrical pore model, are shown in Figure 3. The textural properties of the samples studied, the surface area estimated by different methods (Brunauer–Emmett–Teller (BET), BJH (Barrett, Joyner and Halenda) and NLDFT), as well as the pore volume and pore size distribution by the BJH (determined from the desorption branch of the hysteresis loops) and NLDFT methods are listed in Table 2. The NLDFT method was chosen as more reliable because it provides access to micropores (<2 nm); however, BET and BJH data are also provided for a comparison—the latter allows the contribution of mesoporosity (2–50 nm) to be estimated [56].

The adsorption of the parent Z0 sample is characterized by a combination of isotherms of types I and II, typical for microporous materials. For other samples that do not contain iron, the adsorption branch of the isotherms can be classified as type II-IV, which corresponds to the mode of polymolecular adsorption. The samples exhibit hysteresis in the adsorption/desorption isotherms (IVa according to the IUPAC classification), which indicates the simultaneous presence of both mesopores and micropores, as well as capillary condensation in the mesopores. For samples subjected to alkaline treatment or pre-treatment, the hysteresis is much wider, indicating significantly higher mesopore volume [57].

A significant increase in nitrogen adsorption at high *P*/*P*_0_ values for all the samples evidences the presence of macropores and/or aggregates of disoriented small particles, which is confirmed by SEM, see Figure 2. The shape of the hysteresis loops on the N_2_ adsorption/desorption isotherms is a combination of types H3 and H4 (the IUPAC classification). The presence of H3-type hysteresis points out that mesopores (and macropores) are connected through micropores, similar to what is observed in activated carbons [58]. As can be seen from Table 2, alkaline treatment results in a decrease in surface area due to the formation of mesopores. The pore size distribution obtained from the NLDFT method shows the subsequent appearance of new peaks corresponding to larger mesopores. For samples containing iron, the main feathers of the adsorption/desorption curves are preserved; however, the effect on porosity depends on the alkaline pretreatment: for the non-treated microporous Z0 sample, the porosity decreases, for the sample Z0.2 with a moderate mesoporosity it does not change significantly, while for the sample with expanded mesoporosity Z0.2 after ion-exchange in 1.0 N FeCl_3_, the surface area and pore volume increase sharply due to the subsequent expansion of mesoporosity. Moreover, Z0.4-Fe1.0 exhibits a two-stage desorption branch, indicating the presence of a structure with open pores [59].

### 2.4. UV-Vis Studies

**Figure 4:**
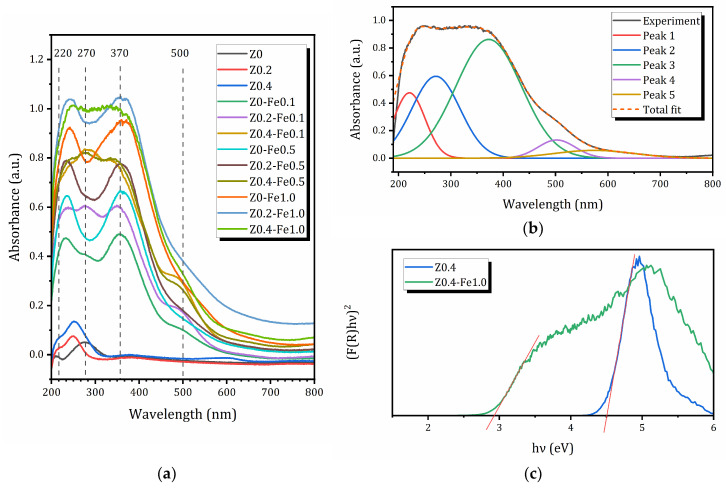
(**a**) UV-Vis spectra of the sample before and after treatment in FeCl_3_ solution; (**b**) decomposition of UV-Vis spectra for the Z0.4-Fe1.0 sample; (**c**) (F(R)hν)^2^ versus photon energy for calculation of bandgap energies for Z0.4 and Z0.4-Fe1.0 samples (the intensities are adjusted for better visualization); red lines show the linear extrapolation of the Kubelka-Munk function.

The coordination environment of iron species in the eight prepared samples was characterized by UV-Vis spectroscopy. The absorption spectra of the studied compounds are shown in Figure 4a. The spectra of the parent micro- and micro/mesoporous materials were used as a reference. The parent compounds Z0, Z0.2, and Z0.4, which do not contain iron, have virtually no UV absorption, whereas the samples containing iron exhibit various absorption bands, the intensity of which strongly depends on both the mesoporosity of the parent compound and the iron content.

All samples exhibit at least four main bands in the UV-Vis spectra, at approximately 220, 270, 370, and 500 nm. An example of the decomposition of the UV-Vis spectrum is shown in Figure 4b. According to several studies of Fe-modified zeolites [16,60,61], the sub-bands at 220 and 270 nm can be associated with isolated Fe^3+^ in tetrahedral and higher coordination (Fe^3+^_T,_ Fe^3+^_O_), respectively. The broader band centered at about 370 nm is usually assigned to Fe^3+^ ions belonging to small oligonuclear Fe_x_O_y_ clusters (Fe^3+^_oli_), while the band centered around 500 nm is attributed to larger iron oxides, such as Fe_2_O_3_ nanoparticles (Fe^3+^_oxi_). As can be seen, Fe_x_O_y_ clusters dominate the zeolite samples under study; however, isolated Fe^3+^ ions are also present, which may isomorphically substitute Al^3+^ in the zeolite framework. The contribution of Fe_2_O_3_ nanoparticles is not very significant compared to other species, and no traces of any iron oxide phases were detected by XRD. Nevertheless, their presence may explain the decrease in specific surface due to the blocking of MFI micropores by nanoparticles of various iron species. The relative intensities of individual bands obtained by deconvolution of the UV-Vis spectra into Gaussian lines, similar to those shown in Figure 4b, are listed in Table 3. Based on the assignment of these peaks, the weight percentages of different Fe^3+^-containing species can be estimated, which are also provided in Table 3.

As can be seen from Table 3, the relative concentration of Fe^3+^_T_ and Fe^3+^_O_ species is not very sensitive to the preparation method, and ranges from 10 to 20%. In all samples, olygonuclear Fe_x_O_y_ complexes predominate (from 38 to 71%), and their concentration increases both with the increase in the mesopore volume of the parent zeolite and with the increase in the concentration of FeCl_3_ solution; meanwhile, iron oxide species show the opposite tendency. Therefore, it has been proven that the creation of mesopores before ion-exchange promotes the formation of olygonuclear Fe_x_O_y_ complexes and isolated Fe^3+^ species.

To find the bandgap energies E_g_, diffuse reflectance spectra of the samples were transformed into coordinates (F·hν)^2^ = f(hν), where F(R) = (1 − R)^2^/2R is the Kubelka–Munk function of a reflection coefficient R. Long-wave absorption edges on the graphs were linearly extrapolated to the intersection with the energy axis and the point found was considered as the optical bandgap energy Eg [62], Figure 4c. The bandgap energies determined using the procedure described above are listed in Table 4.

As can be seen from Figure 4c and Table 4, the microporous sample Z0 is characterized by a rather high bandgap energy of 4.13 eV. The introduced mesoporosity even increases this value up to 4.5–4.6 eV, which allows these materials to function as photocatalysts using only a small part of the near-ultraviolet range with wavelengths up to 310 nm. The insertion of iron leads to a significant reduction in the bandgap; even with an extremely low Fe content (0.9 wt%), E_g_ is 3.06 eV, increasing the absorption maximum. At maximum iron concentrations for each series of modified samples, a shift of the long-wavelength absorption boundary into the visible range (E_g_ ≈ 2.95 eV, λ_max_ ≈ 420 nm) is observed. Thus, the introduction of Fe into the zeolite matrix is an effective approach to expanding the spectral range of activity of these materials, including the potential operation of photocatalysts based on the zeolites under consideration, which is of great importance for the use of not only the ultraviolet but also the visible part of solar radiation.

## 3. Materials and Methods

The parent microporous NH_4_-ZSM-5 zeolite (MFI topology according to International Zeolite Association) with nominal Si/Al ratio 15 was supplied by Zeolyst Int. (Kansas City, KS, USA), CBV 3024E product. The sample was labeled as Z0. To prepare mesoporous ZSM-5, the parent compound was treated in 0.2 M or 0.4 M of NaOH aqueous solution (>99.0% Vekton, Saint-Petersburg, Russia) under stirring (350 rpm) at 65 °C for 120 min, then centrifuged for 10–15 min, washed in distilled water, and dried at 100 °C for 5–6 h. The samples with introduced mesoporosity were labeled as Z0.2 and Z0.4, respectively.

To prepare Fe-exchanged systems, Z0, Z0.2, and Z0.4 samples were treated in aqueous solutions with a concentration of 0.1, 0.5, or 1.0 N of iron (III) chlorate FeCl_3_∙6H_2_O (>99.0% Vekton, Russia) under stirring (500 rpm) at 20 °C for 24 h. After treatment, the samples were washed with deionized water, centrifuged for 15 min, and dried at 100 °C overnight. Hereafter in this paper, samples will be referred as Z*X*-Fe*Y*, where X indicates the concentration of the NaOH solution in the pre-treatment procedure and *Y* denotes the concentration of iron (III) chlorate solution (example: Z0.2-Fe0.5).

The crystal structure of synthetized materials was controlled by X-ray diffraction (XRD) with a Rigaku Miniflex II benchtop Röntgen diffractometer (Tokyo, Japan) with Cu Kα radiation in a 2θ angle range of 3–60° with a step of 0.02°. Phase composition of the materials was controlled using Rigaku PDXL 2.0 software and information resources of the International Centre for Diffraction Data (ICDD). XPS studies were carried out by applying a Combined Auger, X-ray, and Ultraviolet Photoelectron spectrometer Thermo Fisher Scientific (Waltham, MA, USA) ESCAlab 250Xi with AlK radiation (photon energy 1486.6 eV) with total energy resolution of 0.3 eV. The bulk elemental composition was determined using energy-dispersive X-ray fluorescence spectroscopy (EDXRF) in a vacuum using a Shimadzu EDX 800 HS apparatus (Kyoto, Japan).

The morphology of the samples was studied by an optical system integrated into a D8 DISCOVER spectrometer and by scanning electron microscopy (SEM) applying Zeiss Merlin (Oberkochen, Germany) equipped with an energy-dispersive X-ray spectrometer, Oxford Instruments INCAx-act.

N_2_ adsorption isotherms were obtained at 77 K using the Quadrasorb SI 2SI-MP-20 equipment (Quantachrome Instruments, Boynton Beach, FL, USA). Before analysis, the samples were outgassed under vacuum in a FLOVAC Degasser FVD-3 degasser for 3 h at 300 °C. The data processing was performed with QuadraWin software (Version 5.11) (Quantachrome Instruments, Boynton Beach, FL, USA). Pore size distributions and total pore volumes were obtained by Nonlocal Density Functional Theory (NLDFT). Other methods were applied for comparative usage, i.e., the Brunauer–Emmett–Teller (BET) method for surface area estimation and the Barrett–Joyner–Halenda (BJH) method for pore size estimation.

Light absorption of the zeolite-based materials in the ultraviolet and visible region (UV-Vis) was investigated via conventional diffuse reflectance spectroscopy (DRS) performed using a Persee T8DCS spectrophotometer (Auburn, CA, USA) equipped with a SI19-1 integrating sphere. In a typical experiment, ≈50 mg of the powdered material was placed on the quartz glass of a special sample holder. The remaining space in the holder was filled with barium sulfate, which practically does not absorb radiation in the UV-Vis range. After this, the sample holder was placed on one of the windows of the integrating sphere, which collects all the radiation reflected (scattered) by the sample and directs it to the detector. The reflectance spectra R = R(λ) were recorded in the range of 200–800 nm and then transformed into the absorption ones, log(100/R). Peak deconvolution in the absorption spectra obtained was performed using OriginPro 9.5 software (OriginLab, Northampton, MA, USA).

## 4. Conclusions

This study demonstrates that preliminary alkaline modification of ZSM-5 accompanied by the creation of mesopores and subsequent ion-exchange with Fe^3+^ ions allows for targeted regulation of the textural and optical properties of zeolite. The formation of mesopores increases the accessibility of internal channels for hydrated iron ions and promotes the formation of multinuclear Fe_x_O_y_ clusters, while preserving some isolated Fe^3+^ centers capable of replacing Al in the framework. These structural changes significantly affect the optical response: even small amounts of iron noticeably reduce the bandgap energy and expand the absorption spectrum into the visible light region. The results confirm that the combination of alkali treatment and ion-exchange is an effective strategy for creating hierarchically porous Fe zeolites with great potential for photocatalytic applications.

## Figures and Tables

**Table 1:** Chemical composition (atomic ratio) on the surface (XPS) and in the bulk (EDXRF) of the studied samples. For EDXRF Fe weight % is also provided.

Sample	XPS			EDXRF			
	Si/Al	Na/Al	Fe/Al	Si/Al	Na/Al	Fe/Al	Fe (wt%)
Z0	14.39	-	-	10.15	-	-	
Z0.2	9.05	2.31	-	7.77	1.49	-	
Z0.4	5.49	2.2	-	4.96	1.79	-	
Z0-Fe0.1	14.56	-	0.20	9.3	-	0.21	0.9
Z0.2-Fe0.1	14.98	0.44	0.48	8.5	0.28	0.39	1.8
Z0.4-Fe0.1	13.13	0.36	0.98	7.8	0.38	0.92	3.1
Z0-Fe0.5	13.19	-	0.22	9.34	-	0.57	2.5
Z0.2-Fe0.5	12.18	2.09	0.42	8.83	0.11	1.33	5.7
Z0.4-Fe0.5	16.68	0.45	0.84	9.44	0.06	2.61	10.0
Z0-Fe1.0	13.49	0.19	0.69	9.05	-	2.15	8.8
Z0.2-Fe1.0	13.52	0.32	0.61	8.84	0.21	3.36	13.0
Z0.4-Fe1.0	15.36	0.42	1.12	9.29	0.34	8.62	25.0

**Table 2:** Textural properties of the samples before and after treatment in 1.0 N FeCl_3_.

Sample	S_BET_ (m^2^/g)	S_BJH_ (m^2^/g)	S_NLDFT_ (m^2^/g)	V_BJH_ (cm^3^/g)	V_NLDFT_ (cm^3^/g)	D_BJH_ (nm)	D_NLDFT_ (nm)
Z0	409	33	747	0.127	0.24	5	3–7
Z0.2	353	32	635	0.277	0.30	17	15
Z0.4	263	102	362	0.418	0.40	28	6/10/25
Z0-Fe1.0	333	35	574	0.103	0.19	5.5/17	3–7
Z0.2-Fe1.0	349	62	576	0.212	0.27	12	3.5/6/25
Z0.4-Fe1.0	338	135	435	0.434	0.36	28	3.5/6/30

**Table 3:** The relative intensities of the individual UV bands and the content of corresponding Fe^3+^ species for the studied iron-containing samples.

Sample	220 nm		270 nm		370 nm		400–650	
	I (%)	Fe^3+^_T_ (wt.%)	I (%)	Fe^3+^_O_ (wt.%)	I (%)	Fe^3+^_oli_ (wt.%)	I (%)	Fe^3+^_oxi_ (wt.%)
Z0-Fe0.1	18	0.16	19	0.17	38	0.34	25	0.23
Z0.2-Fe0.1	20	0.36	11	0.21	57	1.03	12	0.21
Z0.4-Fe0.1	8	0.26	11	0.35	71	2.16	10	0.32
Z0-Fe0.5	18	0.44	17	0.42	42	1.06	23	0.57
Z0.2-Fe0.5	15	0.86	23	1.3	47	2.7	15	0.83
Z0.4-Fe0.5	8	0.75	19	1.96	62	6.2	11	1.09
Z0-Fe1.0	14	1.25	24	2.1	47	4.12	15	1.33
Z0.2-Fe1.0	19	2.41	14	1.81	50	6.53	17	2.25
Z0.4-Fe1.0	14	3.39	26	6.54	52	12.91	8	2.15

**Table 4:** Bandgap energy (E_g_) and boundary of long-wavelength absorption (λ_max_) for the studied compounds.

Sample	E_g_ (eV)	λ_max_ (nm)
Z0	4.13	300
Z0.2	4.66	266
Z0.4	4.50	276
Z0-Fe0.1	3.06	405
Z0.2-Fe0.1	3.06	405
Z0.4-Fe0.1	3.08	403
Z0-Fe0.5	3.01	412
Z0.2-Fe0.5	3.03	409
Z0.4-Fe0.5	3.04	408
Z0-Fe1.0	2.96	419
Z0.2-Fe1.0	2.96	419
Z0.4-Fe1.0	2.93	423

## Data Availability

The data supporting the reported results are available upon reasonable request.

## Abbreviations

The following abbreviations are used in this manuscript:

BET	Brunauer–Emmett–Teller (method)
BJH	Barrett–Joyner–Halenda (method)
DRS	Diffuse reflectance spectroscopy
EDXRF	Energy-dispersive X-ray fluorescence spectroscopy
NLDFT	Nonlocal density functional theory
SEM	Scanning electron microscopy
XRD	X-ray diffraction
XPS	X-ray photoelectron spectroscopy
